# Vertebral compression fractures: pain relief, progression and new fracture rate comparing vertebral augmentation with brace

**DOI:** 10.1186/s12891-023-07041-1

**Published:** 2023-11-18

**Authors:** Raquel Gutierrez-Gonzalez, A. Royuela, A. Zamarron

**Affiliations:** 1 Department of Neurosurgery, Puerta de Hierro University Hospital, IDIPHISA Manuel de Falla 1, Majadahonda-Madrid, 28222 Spain; 2 Department of Surgery, Faculty of Medicine, Autonomous University of Madrid, Arzobispo Morcillo 4, 28029 Madrid, Spain; 3 Biostatistics Unit. Biomedical Research Institute, Puerta de Hierro University Hospital, IDIPHISA. CIBERESP, Manuel de Falla 1, Majadahonda-Madrid, 28222 Spain

**Keywords:** Fractures, compression, Kyphoplasty, Spinal fractures, Vertebroplasty, Bone Diseases, metabolic

## Abstract

**Background:**

Osteoporotic vertebral compression fracture (VCF) is the third most frequent fragility fracture in the world. Conservative treatment, vertebroplasty, and kyphoplasty are all recognized therapies. However, diagnostic and therapeutic recommendations must be more consistent when comparing clinical guidelines. This study aims to compare the efficacy of vertebral augmentation therapy and conservative management for treating VCFs, the risk of subsequent complications, and the length of hospital stay.

**Method:**

All patients over 50 years old with a diagnosis of thoracic or lumbar VCF without underlying oncological process, treated conservatively or surgically, and consecutively attended at our department from January 2017 to June 2021 were retrospectively selected for analysis. Patients who missed follow-up or died during the first three months were excluded.

**Results:**

A total of 573 cases were selected for analysis. Most patients were treated conservatively (85.3%). Both groups were homogenous regarding epidemiological and clinical features. The median time elapsed to achieve pain relief was significantly lower in the surgical cohort (4.5 vs. 10 weeks, *p* < 0.001), and the proportion of patients reporting pain at the first outpatient visit was also significantly lower with a vertebral augmentation procedure (*p* = 0.004). The new fracture rate and the adjacent level rate did not differ significantly when comparing both treatments, whereas the progression of the diagnosed fracture was more frequent in the conservative group (4.8% vs. 29.7%; *p* < 0.001). The median hospital stay was significantly lower in the conservative group (3 vs. 10 days; *p* < 0.001).

**Conclusion:**

Surgical treatment (vertebroplasty/kyphoplasty) of VCFs was associated with sooner pain relief without an increased risk of new or adjacent fractures. Moreover, the progression of treated fractures was significantly lower in the surgical cohort. The only unfavorable aspect was the more extended hospital stay compared with the conservative treatment group.

## Background

Osteoporotic vertebral compression fracture (VCF) is the third most frequent fragility fracture in the world, resulting in many cases in a long-lasting, painful, and disabling condition [1]. The risk of vertebral fracture increases in women over 50 years old, just as the risk of osteoporosis does. Thus, the risk of suffering a VCF in a 50-year-old woman reaches 40% in her lifetime. On the contrary, the risk of proximal femoral fracture is higher in patients over 75 years old [2]. Moreover, VCF incidence is 10 times higher than femoral fracture incidence and may not be related to falls, unlike other site fractures, which are secondary to trauma [2]. While fall prevention focuses on active life, exercise, vitamin D intake, and environment-home adaptation, fracture prevention has yet to be well established [2]. When considering secondary prevention of osteoporotic VCF, different drugs such as bisphosphonates, parathyroid hormone, denosumab, and selective estrogen receptor modulators have been demonstrated useful [3].

Conservative treatment (bed rest, brace, analgesics), vertebroplasty, and kyphoplasty are all recognized therapies for managing VCF. There is an increasing number of systematic reviews of the literature and meta-analyses that highlight the efficacy of vertebral augmentation through percutaneous vertebroplasty or kyphoplasty for managing VCF compared with conservative treatment [4–6]. However, the Cochrane review concluded that this effect might be overestimated and, therefore, the real impact may be lacking [7]. According to several studies, vertebroplasty and kyphoplasty have been associated with better pain relief when compared with non-invasive management [4–6, 8–10]. Moreover, they have been proven beneficial in cancer-related VCFs [11]. These surgical therapies have also been associated with better physical function and quality of life [4, 5], even though other data disagree [9]. Finally, lower mortality excess has been attributed to surgery in fragile patients [12]. Nevertheless, diagnostic and therapeutic recommendations are usually inconsistent when comparing different clinical guidelines [13]. Thus, the American Academy of Orthopedic Surgeons has advised against vertebroplasty for treating osteoporotic VCFs [13].

The aim of this study is to compare the efficacy of vertebral augmentation therapy and conservative management for the treatment of VCF, the risk of subsequent complications (not only new vertebral fracture but also treated vertebra re-fracture), and the length of hospital stay.

## Methods

A single-center, retrospective cohort study was designed to assess the presence or absence of pain, and the incidence of new fractures or the progression of the known fracture in VCFs when comparing percutaneous vertebral augmentation therapy (vertebroplasty/kyphoplasty) and conservative management (orthosis). The study was approved by the local Ethics Committee of Puerta de Hierro University Hospital (reference 157/21) and was conducted in accordance with the 1964 Helsinki Declaration and its later amendments or comparable ethical standards. This study is reported following the STROBE guidelines [14].

### Patient selection

All patients over 50 years old diagnosed with acute thoracic or lumbar VCF at levels T5 to L5, in the absence of underlying oncological process, treated conservatively or surgically and consecutively attended at our department from January 1st of 2017 to June 30th of 2021 were retrospectively selected for analysis. Patients who missed follow-up and those who died during the first three months following diagnosis were excluded. Patients having undergone percutaneous vertebroplasty or kyphoplasty were allocated to the surgical cohort, while those managed with a brace were assigned to the conservative cohort. In those cases with patients presenting a cross-over in the treatment, only the first therapy was considered since some variables, such as pain assessment, could be interfered with.

Individual surgeon’s preference was the main factor for therapy assignment. Patient preference and comorbidity were also determinants in a minority. In our department, half of neurosurgeons treat these fractures with a brace, whereas the remaining half offer the patient the possibility to treat the fracture with a brace or surgery. Then, the patient decides. Only in a few specific cases, according to patients’ features, the doctor recommends only vertebral augmentation.

Non-surgical management consisted of analgesics and a back brace whenever the patient was incorporated -sitting or standing- until the clinician’s decision. The standard of care in our hospital involves bracing for all patients managed conservatively. This includes different types of devices, but the most frequently used are the Jewett brace (T5-T10 levels) and thermoplastic thoracolumbar or lumbosacral orthosis (T10-L5 levels).

### Dependent variables

Pain relief was assessed by the presence or absence of pain as referred by the patient at the first outpatient visit (8 or 12 weeks of follow-up) as well as the time interval with pain (number of weeks).

A new fracture was defined as a fracture in a different vertebra from the treated one, which could be adjacent or distant to it.

Progression fracture was defined as the increase in the height loss of the vertebral body measured in the sagittal plane using plain X-ray, computed tomography, or magnetic resonance image (MRI).

The length of brace use was measured in weeks. This variable was included since many patients complain about the discomfort of wearing the orthosis.

Hospital stay was defined as the length of stay (number of days) at the hospital, including prior and after treatment, whichever this was.

### Independent variables

Epidemiological, clinical, diagnostic, and therapeutic variables were registered in the database, including gender, age, history of cancer, chronic steroid use, history of previous vertebral fracture, prior diagnosis of osteoporosis, active use of anti-osteoporosis drugs (calcium, D vitamin, bisphosphonates, among others), mechanism of the fracture, spinal segment involved, multiple fractures at diagnosis, presence of spinal tenderness and length of follow-up.

### Statistical analysis

Database information was processed and analyzed employing StataCorp. 2019 (Stata Statistical Software: Release 16. College Station, TX: StataCorp LLC). Numerical variables represented by the mean and standard deviation (SD) were contrasted with the Student-T test, whereas those represented by the median (percentiles 25 and 75 as dispersion measure) were contrasted with the Mann-Whitney U test. The Chi-square test was used in categorical variables and absolute and relative frequencies as the description measure. All percentages were calculated per patient. The considered level of significance was 5%. All p-values were based on two-tailed tests of significance. 

## Results

A total of 687 consecutive fractures were recorded. Incomplete follow-up excluded 85 cases, and another 25 patients died during the first three months after diagnosis. Four patients with initial conservative management required vertebral augmentation due to poor fracture evolution, so the second procedure was excluded. Then, 573 cases were finally analyzed. Data showed female prevalence (71.7%) and mean age at diagnosis of 74.5 (SD 10.3) years old. The baseline characteristics of both cohorts are summarized in Table 1. Most patients were treated conservatively (85.3%), and no adverse events happened after surgical treatment.

**Table 1:** Comparison of baseline characteristics

VARIABLE	OVERALL (n = 573)	CONSERVATIVE TREATMENT (n = 489)	SURGICAL TREATMENT (n = 84)	*p* value
**Mean Age, yr (SD)**	74.5 (10.3)	74.6 (10.4)	73.8 (10.0)	0.497
**Gender Female, n (%)**	411 (71.7)	351 (71.8)	60 (71.4)	0.947
**History of cancer, n (%)**	115 (20.1)	96 (19.3)	19 (22.6)	0.528
**Chronic steroid treatment, n (%)**	77 (13.4)	67 (13.7)	10 (11.9)	0.656
**Previous fracture, n (%)**	163 (28.5)	128 (26.2)	35 (41.6)	**0.004**
**Osteoporosis-diagnosis, n (%)**	195 (34.0)	160 (32.7)	35 (41.7)	0.110
**Osteoporosis-therapy, n (%)**	180 (31.7)	150 (31.0)	30 (35.7)	0.390
**Mechanism of fracture, n (%)**				0.326
*Spontaneous*	*137 (24.0)*	*115 (23.5)*	*22 (26.2)*	
*Fall*	*346 (60.5)*	*293 (59.9)*	*53 (63.1)*	
*Overexertion*	*59 (10.3)*	*51 (10.4)*	*8 (9.5)*	
*High-energy trauma*	*30 (5.2)*	*29 (5.9)*	*1 (1.2)*	
**Spine segment, n (%)**				0.893
*Dorsal*	*219 (38.2)*	*186 (38.0)*	*33 (39.3)*	
*Lumbar*	*292 (51.0)*	*251 (51.3)*	*41 (48.8)*	
*Dorsal & lumbar*	*62 (10.8)*	*52 (10.6)*	*10 (11.9)*	
**Posterior wall injury (type A3 & A4 AOSpine classification), n (%)**	121 (22.3)	96 (20.9)	25 (29.8)	0.073
**Multiple level, n (%)**	134 (23.4)	110 (22.5)	24 (28.6)	0.224
**Midline spine tenderness, n (%)**	382 (72.3)	315 (70.6)	67 (81.7)	**0.039**
**Median follow-up, wk (IQR)**	20.5 (12; 29)	21 (13; 29)	15 (8; 27)	0.155

**IQR**: interquartile range; **SD**: standard deviation; **wk**: week; **yr**: year

The median time elapsed until achieving pain relief was significantly lower in the surgical cohort (4.5 vs. 10 weeks, *p* < 0.001), and the proportion of patients reporting pain at the first outpatient visit (8–12 weeks from diagnosis) was also significantly lower when they underwent a vertebral augmentation procedure (*p* = 0.004). The new fracture rate and the adjacent level rate did not differ significantly after both treatments. In contrast, progression or re-fracture of the diagnosed fracture was significantly more frequent in the conservative cohort (29.7% vs. 4.8% in the surgical group, *p* < 0.001). The median time elapsed until a progression of the fractured vertebra was diagnosed (due to symptoms or in a radiological follow-up) was 9 weeks, and no difference was observed between both treatment groups. In contrast, the median time elapsed until a new fracture of a different vertebra was diagnosed was slightly inferior in the conservative group (9 weeks) compared to the surgical group (11 weeks). Finally, the median hospital stay was significantly lower in the conservative group (3 vs. 10 days, *p* < 0.001), whereas the median time interval with a brace was significantly lower in the surgical cohort (0 vs. 12 weeks, *p* < 0.001). Table 2 summarizes the outcome results.

**Table 2:** Comparison of outcome variables

VARIABLE	OVERALL (n = 573)	CONSERVATIVE TREATMENT (n = 489)	SURGICAL TREATMENT (n = 84)	*p* value
**Pain at first outpatient visit (8–12 wk), n (%)**	178 (31.5)	163 (33.8)	15 (18.1)	**0.004**
*Median time to pain relief, wk (IQR)*	10 (7; 14)	10 (8; 15)	4.5 (1; 8.25)	**< 0.001**
**New fracture, n (%)**	58 (10.1)	48 (9.8)	10 (11.9)	0.558
*Adjacent level*	*23 (48.9)*	*20 (41.7)*	*3 (30.0)*	*0.477*
*Median time to new fracture, wk (IQR)*	*9 (6; 14.75)*	*9 (6; 14.25)*	*11 (3.25; 20)*	*0.251*
**Fracture progression, n (%)**	149 (26.0)	145 (29.7)	4 (4.8)	**< 0.001**
*Median time to progression, wk (range)*	*9 (7; 13)*	*9 (7; 13)*	*9 (5; 15.75)*	*0.841*
**Mean time interval with brace, wk (IQR)**	12 (8; 16.75)	12 (9; 18)	0 (0; 0)	**< 0.001**
**Median hospital stay, dy (IQR)**	4 (2; 9)	3 (2; 8)	10 (7; 13)	**< 0.001**

**dy**: day; **IQR**: interquartile range; **wk**: week

## Discussion

According to the results, vertebral augmentation treatment of VCFs was associated with sooner pain relief in the absence of an increased risk of new or adjacent fractures. Moreover, the progression of treated fractures was significantly lower in the surgical cohort. The only unfavorable aspect was the more extended hospital stay compared with the conservative treatment group.

Clinical management of VCFs remains controversial despite the increasing evidence of vertebral augmentation safety and efficacy in treating VCFs [13]. The comparison of percutaneous vertebroplasty with a sham procedure demonstrated a significant reduction in pain regarding the preoperative condition and the analgesic intake in both groups. Still, no difference was observed between both techniques [7, 15]. When comparing vertebroplasty with kyphoplasty, both procedures seem to be beneficial for pain relief and daily physical function [16], but results show similar clinical outcomes between both techniques in some cases [17, 18], whereas other studies outline the superiority of kyphoplasty [19]. Most studies quantify pain decrease with the visual analog scale [4, 6]. The results hereby obtained also show sooner relief with surgical management. Radiological results have also been reported (kyphotic angle, vertebra height), with consensus on the superiority of kyphoplasty over vertebroplasty [17–19]. Finally, cement leakage has been extensively demonstrated to be inferior with kyphoplasty [16–18].

An important outcome widely reported after VCF diagnosis is the presence of a new vertebral fracture, which may be distant or adjacent to the primary one. Even though occasional studies report a reduced rate with vertebroplasty [5], most evidence points out no significant difference between vertebral augmentation procedures and conservative treatment [7, 8, 10, 20–24], a fact that is confirmed by the results now detailed. No difference has been described when comparing vertebroplasty and kyphoplasty [19, 25]. The time elapsed until a new vertebral fracture diagnosis is variable, but no difference has been reported among different treatment groups [26], as it occurs in the present research. Risk factors for new fracture following vertebroplasty have been identified (low bone mineral density, high spinal deformity index, low fracture age, thoracolumbar localization, vicinity to the treated level), and the presence of intradiscal cement leakage (and the volume of cement) has been considered strongly associated with it [27]. The limit between predisposition to this condition and the natural history of the disease must be established.

Progression or re-fracture of the treated vertebra has received less attention in the meta-analyses that compare vertebral augmentation with conservative therapy but has been occasionally analyzed [26]. The reported incidence varies from less than 1–63% [28–31], with a cumulative rate of 10% [29]. In the series hereby reported, almost 30% of patients that were managed conservatively showed vertebral re-fracture, which may be associated with worse pain control or more extended bracing, and that must be considered when choosing any therapy. Thus, the re-fracture rate was significantly higher when a brace was used rather than following vertebral augmentation. These results are similar to those observed in a randomized clinical trial, although in this latter case, rates were even higher (41% when conservative management vs. 12% following vertebroplasty) [26]. This variable has also been compared between vertebroplasty and kyphoplasty, and the latter procedure seems to be associated with higher rates of progressive height loss [32].

A recent meta-analysis identifies risk factors for vertebral re-collapse following vertebroplasty: thoracolumbar junction fractures, preoperative intravertebral cleft, and solid lump cement distribution pattern [30]. A previous meta-analysis that also includes kyphoplasty procedures adds two more factors: preoperative severe kyphotic deformity and higher vertebral height restoration [29]. No similar meta-analysis has been performed in the case of conservative management. Still, the research published to date identifies age, thoracolumbar fracture, AOSpine type fracture, or a linear black area at MRI as predictive factors for progressive collapse [33–36]. Even though posterior wall injury is a relative contraindication for vertebral augmentation due to cement leakage risk, the proportion of A3 or A4 fractures (according to the AOSpine classification) was higher in the surgical group compared with the conservative management one, but no statistically significant difference was observed between both cohorts.

Hospital length of stay is another variable that has been vaguely considered when comparing VCF treatments and remains uncertain [37]. The prevalence of VCF hospitalization has been demonstrated to be higher in the case of ankylosing spondylitis when compared with other rheumatoid diseases or the general population [38]. However, almost all patients attending at our center are usually admitted until therapy is guaranteed (surgery or brace). The differences observed in this study are then related to the delay in surgery scheduling rather than postoperative care (which is often short), and it is a factor that provokes discomfort in patients since they undergo bed rest until surgery is performed. An extended hospital stay may also be related to higher costs, at least initially, since the cost-effectiveness of vertebral augmentation is strongly associated with the assumed mortality benefit. Some data point out that vertebroplasty or kyphoplasty may lead to lower mortality. However, a causal link has not been definitively established [37].

Finally, many patients have reported discomfort with the use of the brace. Even though it is a temporary measure, it may interfere with the quality of life perceived by the patient. The mean length of use was significantly higher in non-surgical patients since it is exceptional to employ it after surgery. However, no conclusion can be inferred since the quality of life has yet to be registered.

Two main limitations must be outlined. The first one refers to the retrospective design of the study. This fact prevented quantifying pain relief using a visual analog scale since many surgeons don’t register this information routinely at follow-up visits. Then, a qualitative method was chosen for pain evaluation, relying on analgesic drug intake. The second one responds to the length of follow-up. It is noteworthy that follow-up was shorter following a vertebral augmentation procedure, a fact that may be explained since these patients had better (and sooner) control of pain. In contrast, those who received conservative management presented long-lasting pain and a higher re-fracture rate of the treated vertebra, which frequently involved more prolonged brace use and longer follow-up.

The second one responds to the length of follow-up. It is noteworthy that follow-up was shorter following a vertebral augmentation procedure, a fact that may be explained since these patients had better (and sooner) control of pain. In contrast, those who received conservative management presented long-lasting pain and a higher re-fracture rate of the treated vertebra, which frequently involved more prolonged brace use and longer follow-up.

## Conclusions

Vertebral augmentation treatment (vertebroplasty/kyphoplasty) of VCFs in patients over 50 was associated with sooner pain relief, but hospital stay was more extended than in the conservative treatment group. It also entailed a lower risk of progression of treated fractures, but the risk of new fractures did not differ from conservative management with a brace.

Vertebroplasty or kyphoplasty must be considered in the management of VCFs in the elderly, particularly in fragile patients, since it may entail better pain control and earlier mobilization, avoiding needless brace.

## Data Availability

The datasets generated and/or analyzed during the current study are available from the corresponding author on reasonable request.

## Abbreviations

dy: Day
IQR: Interquartile range
MRI: Magnetic resonance image
SD: Standard deviation
VCF: Vertebral compression fracture
wk: Week
yr: Year
